# Soil microbes and associated extracellular enzymes largely impact nutrient bioavailability in acidic and nutrient poor grassland ecosystem soils

**DOI:** 10.1038/s41598-022-16949-y

**Published:** 2022-07-23

**Authors:** Khululwa Ndabankulu, Samson O. Egbewale, Zivanai Tsvuura, Anathi Magadlela

**Affiliations:** 1 School of Life Sciences, University of KwaZulu-Natal Westville Campus, Private Bag X54001, Durban, 4000 South Africa; 2 Centre for Functional Biodiversity, School of Life Sciences, University of KwaZulu-Natal, Pietermaritzburg Campus, Private Bag X01, Scottsville, 3209 South Africa

**Keywords:** Grassland ecology, Microbial ecology

## Abstract

Understanding the role of soil microbes and their associated extracellular enzymes in long-term grassland experiments presents an opportunity for testing relevant ecological questions on grassland nutrient dynamics and functioning. Veld fertilizer trials initiated in 1951 in South Africa were used to assess soil functional microbial diversity and their metabolic activities in the nutrient-poor grassland soils. Phosphorus and liming trials used for this specific study comprised of superphosphate (336 kg ha^−1^) and dolomitic lime (2250 kg ha^−1^) (P + L), superphosphate (336 kg ha^−1^) (+ P) and control trials. These soils were analyzed for their nutrient concentrations, pH, total cations and exchange acidity, microflora and extracellular enzyme activities. The analysed soil characteristics showed significant differences except nitrogen (N) and organic carbon (C) concentrations showing no significant differences. P-solubilizing, N-cycling and N-fixing microbial diversity varied among the different soil treatments. β-glucosaminidase enzyme activity was high in control soils compared to P-fertilized and limed soils. Alkaline phosphatase showed increased activity in P-fertilized soils, whereas acid phosphatase showed increased activity in control soils. Therefore, the application of superphosphate and liming influences the relative abundance of bacterial communities with nutrient cycling and fixing functions which account for nutrient bioavailability in acidic and nutrient stressed grassland ecosystem soils.

## Introduction

Grassland ecosystem occupies 40% of the world’s biomes and accounts for 69% of global agricultural productivity which is vital for ecosystem services and climate regulations^1^. This ecosystem plays a crucial role in increasing food security and provides economic livelihood for about 37% of the world's population^2^. Despite, being a global reservoir of biodiversity, carbon storage, water supply and regulation, pollination and a host of cultural services, material and non-material essentials for mankind, its productivity is mainly influenced by climate and inherent soil properties whose continued existence is dependent on proper management practices^1^.

However, soil characteristics of managed grassland establishment with its increased productivity have been linked to relatively high sand and silt and low clay contents, moderate drainage, friable consistency, small aggregates, slightly acidic condition, and poor nutrient levels^3^. Acidic soils formed as results of such activities are associated with high cation concentrations such as Al and Fe which then form insoluble complexes with soil P, thus reducing available P for plant assimilation^4–6^. Fertilization, tillage and other forms of agricultural practices are the common techniques often employed in controlling such problems confronting soil properties and changes in soil quality. Crews and Peoples (2004) suggested that amending soil acidity in ecosystems would require an estimated 4.5 million tons of annual application of lime. However, the present annual estimate of lime production and sales is around one million tons^7^. Therefore, soil acidity is likely to become an increasing problem in ecosystems worldwide including South Africa. In these ecosystems, soil microbes assist in nutrient cycling, thus increasing soil nutrients for plant uptake^8^. These soil microbes include rhizobia and *Bacillus* amongst other bacteria genera^9^.

Soil microflora plays a vital role in the overall functioning of grassland ecosystems^10^; however, soil microbial functioning, diversity and composition are affected by soil pH^11^. Since most microflora inhabits the upper part of the soil profile, where the largest portion of organic deposits, air and water are accessible, disturbances from fertilizer application, tillage, contamination and crop rotation are major influencers of microbial communities with their associated metabolic pathways and the organic horizon with significant consequences on the soil ecosystem^12^. For example, a meta-analysis showed that N addition could reduce soil microbial diversity by an average of 15% of its total soil microbial biomass^13^ while intermittently altering the soil pH^14,15^. While lime (Ca(OH)_2_) application has been implicated in the alteration of the composition of microbial communities and their biological process including microbial activities and carbon mineralization^16^. Yin et al.^17^ similarly revealed how liming marginally alters soil surface bacterial communities, from the families Cytophagaceae, Flavobacteriaceae, Intrasporangiaceae, Phyllobacteriaceae, Propionibacteriaceae, Psudomonadaceae, Sphingobacteriaceae but could not establish any correlation between liming, soil depth and location. Conversely, a recent study revealed that the effects of liming on bacterial community richness or diversity were influenced more by soil location than lime^18^.

Extracellular soil enzymes function as mediators and catalysts in soil biochemical reactions, such as nutrient mineralization and cycling, decomposition and soil organic matter formation^19^. Enzymes such as phosphatases solubilize insoluble cation-bound P complexes, making them available for plant uptake^20^. The secretion and increased activity of enzymes such as phosphatases are how some microbes and plants respond to soil acidity and P deficiency^20,21^. In addition, enzymes such as glycosidases are a group of carbon (C) cycling enzymes that play a crucial role in breaking low molecular weight carbohydrates and are the primary energy source for soil microorganisms^21^. Glycosidase, a-galactosidase, known as cellobiose, catalyses the hydrolysis of the disaccharides, a-d-Galatopyranosides, in soils^22^. β-glucosaminidase is a crucial enzyme involved in the hydrolysis of N-acetyl-b-d-glucosamine^23,24^ residues from the terminal non-reducing ends of chitooligosaccharides^25^. This hydrolysis is considered necessary in C and N cycling in soils. Soil pH also influences soil enzyme activities, such as laccase^26^, peroxidase^27^, N-acetyl-β-d-Glucosaminidase^24^ and β-d-Cellobioside^28^.

We hypothesized that the different types of fertilization regimes could affect nutrient levels in the soil which subsequently influence its metabolic processes and microbial diversity and community structure. Although it has been established that P addition to grassland ecosystem soils only alters its plant community and its productivity such as legume biomass increment^29^, and while soil surface liming has been reported to influence an increase in the relative abundance of some bacteria taxa belonging to families Cytophagaceae and Flavobacteriaceae (Phylum Bacteroidetes), A4b (Phylum Chloroflexi), and Opitutaceae (Phylum Verrucomicrobia)^18^. However, an extensive understanding of the impacts of P-fertilization in combination with liming on soil microbial communities and associated enzyme activities is still limited. To bridge this gap, we seek to investigate the effects of long-term phosphorus (P) fertilization and liming on soil microbe diversity and extracellular soil enzyme activities in acidic and nutrient-poor soil collected from long-term grassland fertilization trial plots at Ukulinga Research Farm, University of KwaZulu-Natal, KwaZulu-Natal province, South Africa.

## Materials and methods

### Study area and experimental design

Soil samples were collected at the Grassland/Veld Fertilizer Trials (VFT) at Ukulinga Research Farm (29° 37′ S: 30° 16′ E, 840 masl), University of KwaZulu-Natal in Pietermaritzburg, South Africa. The site has an altitudinal gradient range between 838 to 847 m above sea level^30^, with annual precipitation and annual temperature of 838 mm and 18 °C respectively^31^. The soil taxonomy according to Westleigh classification showed that it is a shale derived soil with characteristics of relatively infertile and acidic. This site vegetation is dominated by *Hyparrhenia hirta* L. and *Themeda triandra* (Forssk.) with scattered leguminous trees such as *Vachellia sieberiana* DC and *Vachellia nilotica* (L.) P.J.H. Hurter & Mabb^32^. The VFT was initiated in 1951 through the manipulation of N, P and lime (L) without any fire or large herbivore disturbances and is the longest-running field experiment in Africa^33^.

The UGNE involves the manipulation of nitrogen (N), phosphorus (P) and lime within 96 plots and each plot is 9.0 × 2.7 m in size with a 1 m spacing between plots for the period 1951–2019. The experiment was laid out as a split-split-plot design, with fertilizer type as the main-plot factor with the four rates randomly assigned to four main plots in each of three complete replicate blocks, application periods (yearly) as the sub-plot (or split-plot) factor with the three application periods randomly assigned to three sub-plots within each main plot and application periods as the sub-sub-plot (or split-split-plot) factor with the three application periods randomly assigned to individual sub-sub-plots within each sub-plots. The fertilizer application is as follows; firstly, the two forms of nitrogen applied were limestone ammonium nitrate and ammonium sulphate (henceforth LAN and AS, respectively). Four levels of N fertiliser were applied annually on plots 0 (control), 7.1, 14.1, and 21.2 g m^−2^ for both LAN and ASU. In addition, the N treatments were applied either alone or in combination with P and L Both LAN and ASU were applied twice a year in October and December per plot. Secondly, in terms of P addition, the application was in the form of super-phosphate at two levels 0 (control) and 33.6 g m^−2^. Phosphorus was applied once a year in October. Thirdly, the lime treatments were applied every five years at two levels 0 (control) and 225 g m^−2^^34,35^, with the last application being in 2016. Noteworthy the long-term VFT objective was to increase the productivity of fodder and fodder nutrient compositions like crude protein content and that informs the choice of nutrient addition (N, P and liming).

### Experimental soil and soil nutrient analysis

Experimental plots fertilized with superphosphate (336 kg ha^−1^) applied once a year and dolomitic lime (2250 kg ha^−1^) applied every 5 years (P + L), superphosphate (336 kg ha^−1^) applied once a year (+ P) and soils with no superphosphate and no dolomitic lime (control) were used as three soil treatments for this study. In each treatment site, 10 random points were chosen. In each of these 10 points, 10 sub-points were chosen and a hole of 15 cm depth were dug. The 10 cm depth was estimated with a measuring tape. This depth is considered the portion of soil in closer contact with roots and where maximum microbial activity is expected; the collected soil in each point (10 sub-points) was transferred to a bucket and thoroughly mixed. In total 10 compound samples were collected per site. A portion of each compound soil sample was stored in sterile plastic bags in a refrigerator at 4 °C until chemical and biological analyses were conducted. Soil samples of 50 g with five replicates were sent for P, N, K, pH, acidity exchange and total cation analysis at the KwaZulu-Natal Department of Agriculture and Rural Development’s Analytical Services Unit, Cedara, South Africa. Ground soil samples were analyzed for total N with the Automated Dumas dry combustion method using a LECO CNS 2000 (Leco Corporation, Michigan, USA) and pH (using a KCl solution). Phosphorus and K in the soil samples were measured using atomic absorption method. This involved the extraction of 2.5 mL soil solution with 25 mL ambic-2 solution at pH of 8. The mixture was stirred at 400 rpm for 10 min using a multiple stirrer and filtered using Whatman No.1 paper. An additional five soil subsamples (250–300 g) from each treatment were used for microbial identification and enzymatic analysis.

### Soil-borne bacteria

A three-fold serial dilution was conducted on 10 g of soil samples and 100 μL of each dilution was used to inoculate nutrient agar (NA) plates. The inoculated plates were incubated at 37 °C for 18 h and distinct bacterial colonies were enumerated using the colony-forming unit (CFU) method^36^.

#### Bacterial DNA extraction

Bacterial DNA was extracted using a modified boiling procedure described by Akinbowale^37^. Bacterial colonies (≤ 5) picked off NA plates were suspended in 70 μL MilliQ H_2_O, boiled in a water bath at 100 °C for 10 min and placed on ice for 5 min. The suspension was centrifuged at 13,817×*g* in a micro-centrifuge (Spectrafuge 16 M, Labnet) for 5 min and the supernatant (~ 50 μL) was transferred to sterile Eppendorf tubes.

#### DNA amplification, sequencing and identification

The extracted bacterial DNA was amplified using the 16S ribosomal RNA gene primers: 63F (5′-CAG GCCTAACACATGCAAGTC-3′) and 1387R (5′-GGGCGGTGTGTACAA GGC-3′). The PCR mixtures consisted of 10 μL DNA, 5 μL of 10 X reaction buffer, 2 μL 25 mM MgCl_2_, 2.5 μL of each primer, 0.25 μL of Taq DNA polymerase, 1 μL of 10 mM dNTP and volume made up to 50 μL with MilliQ H_2_O. A T100 Thermal Cycler (BioRad, USA) was used for amplification with the initial denaturation at 94 °C for 2 min followed by 30 cycles of denaturation at 92 °C for 30 s, then annealing at 56 °C for 45 s and elongation at 75 °C for 45 s with a final elongation at 75 °C for 10 min. The PCR products were resolved on 1.0% (w/v) agarose gels (Seakem, Lonza, USA) and visualized after staining with ethidium bromide (0.5 μg mL^−1^) using the Chemigenius Bio-imaging System (Syngiene, England). Positive amplicons (~ 1324 bp) were excised and sequenced at Inqaba Biotechnical Industries and the sequences were compared against the GenBank database. Homologues were identified using the Blastn program at the National Center for Biotechnology Information (NCBI) (https://blast.ncbi.nlm.nih.gov/Blast.cgi).

### Measurement of biodiversity

The biodiversity measurements were based on total CFU g^−1^, percentage relative abundance, relative density, isolation frequency, Shannon–Wiener index of diversity (H), Simpson’s index of dominance (D), R_margalef_ and E_pielou_.

#### Microbial population count

Soil samples were subjected to serial dilution and spread on selective media agar plates in accordance to standard solid plating techniques. P-solubilizing bacteria were isolated and enumerated using Pikovskaya’s agar plates containing tricalcium phosphate (TCP) as the phosphate source. The N cycling bacteria was enumerated on Simmons Citrate agar plates which contain citrate as the carbon (C) source and inorganic ammonium salts as the only source of N while that of the N fixing bacteria and cycling bacteria carried out on Jensen’s media agar (N free media). Each selective media plate was replicated in triplicate and incubated at 30 °C for 5 days. The microbial population were expressed as CFU g^−1^.

#### Ecological parameters (structural indices)

Based on 16S rRNA gene sequences, the bacterial community richness and diversity was analyzed based on total CFU g^−1^, percentage relative abundance, Dominance D, Shannon–Wiener index of diversity (H), Simpson index of dominance (D), R_margalef_, E_pielow_ and homogeneity (equitability)(J).

### Soil enzyme activity

The activities of N and P cycling enzymes (β-glucosiamindase, acid phosphatase and alkaline phosphatase) were assayed using a fluorescence-based method described by Jackson^38^ and expressed in units of nmol h^−1^ g^−1^. In brief, five soil samples were homogenized at low speed in 50 mL milliQ H_2_O for 2 h at 4 °C. The supernatants were transferred into black 96-well microplates prior to adding the respective substrates. The sample run consisted of 200 μL soil aliquot plus 50 μL substrate and incubated alongside reference standards (200 μL buffer + 50 μL standard), quench standards (200 μL soil aliquot + 50 μL standard), sample controls (200 μL soil aliquot + 50 μL buffer), negative controls (200 μL buffer + 50 μL substrate) and blanks (250 μL buffer). The reaction was stopped with 0.5 M NaOH after a 2 h incubation at 30 °C. Thereafter, fluorescent absorbance was measured at 450 nm on a Glomax Multi Plus microplate reader (BioTek, USA). Before the determination of acid phosphatase activity both the buffer and standards were adjusted to pH 5.

Nitrate reductase (NR) activity was determined according to a slight modification of the method of Goyal^39^; Pavlovic^40^. Briefly, 5 g of soil was added to a solution consisting of 1 mL of 25 mM KNO_3_, 4 mL of 0.9 mM 2,4-dinitrophenol and 5 mL of milliQ H_2_O in a sealed 50 mL centrifuge tube. The mixture was then thoroughly mixed before being incubated at 30 °C in the dark for 24 h. After incubation, 10 mL of 4 M KCl solution was added to each sample, mixed briefly and passed through Whatman number 1 filter paper.

The enzymatic reaction was initiated by adding 500 μL of filtrates with 300 μL of 0.19 M ammonium chloride buffer (pH 8.5) and 200 μL of a colour reagent (1% sulfanilamide in 1 N HCl and 0.02% N-(1-naphthyl) ethylenediamine dihydrochloride (NEDD) before incubating in the dark at 30 °C for 30 min. The absorbance was measured at 520 nm using an Agilent Cary 60 UV–Vis spectrophotometer (Agilent, USA). The amount of nitrite (NO_2_^−^) liberated into the medium was extrapolated from a prepared standard curve with KNO_3_. Nitrate reductase activity was expressed as 0.1 μmol h^−1^ g^−1^.

### Data analyses

IBM SPSS statistics for windows v. 24 was used to test for differences in macro, intermediate and micro-nutrients, as well as pH, exchange activity, total cation and soil enzyme activity in the three treatments soils of VFT farm, using one-way analysis of variance (ANOVA). Where the ANOVA showed significant differences between treatments, a Tukey’s post hoc test was used to separate the means at a significance level of 0.05.

## Results

### Soil nutrient concentrations and relative acidity

The analyzed fertilization trial soils (P + L, + P and control) were acidic, with pH ranging from 4.628 to 6.332 (Table 1). Statistical differences were observed among soil nutrition between P + L, + P and control fertilization trials (Table 1). P + L soils had the highest P concentration and control soils had the lowest P concentration. N concentration was higher in + P soils followed by P + L soils and control soils had the lowest N concentration. Potassium (K) concentration was significantly higher in + P soils and lowest in control soils, while magnesium (Mg) concentration was highest in control soils and lowest in + P soils. Organic C concentration showed no significant difference between the soil treatments (Table 1). P + L soils had the highest Ca concentration and control had a significantly lower Ca concentration.

**Table 1:** Soil nutrients concentrations, total cations, exchange acidity and pH in soils supplemented with phosphorus and lime, soils rich in phosphorus and control soils collected at Ukulinga farm, KwaZulu-Natal.

Parameter	P + L	+ P	Control
**Soil nutrients (mmol g**^**−1**^**)**			
P	0.292 ± 0.016^a^	0.256 ± 0.012^a^	0.083 ± 0.005^b^
N	0.198 ± 0.004^a^	0.201 ± 0.002^a^	0.196 ± 0.008^a^
K	4.219 ± 0.674^a^	4.234 ± 0.681^a^	2.531 ± 0.335^b^
Organic C	3.558 ± 0.054^a^	3.711 ± 0.025^a^	3.691 ± 0.166^a^
Ca	63.252 ± 1.774^a^	35.158 ± 0.676^b^	30.789 ± 1.538^c^
Mg	19.393 ± 1.877^a^	15.194 ± 0.588^b^	19.685 ± 1.186^a^
**Relative acidity**			
Exchange acidity (cmol L^−1^)	0.419 ± 0.252^a^	0.238 ± 0.054^b^	0.133 ± 0.022^c^
Total cations (cmol L^−1^)	25.906 ± 1.689^a^	27.138 ± 1.295^b^	27.076 ± 2.597^c^
pH	6.33 ± 0.06^a^	4.65 ± 0.01^b^	4.63 ± 0.03^b^

Values are expressed as means ± SE, n = 5. In each row, different letters represent significant differences based on Tukey’s Post Hoc statistical analysis. (*p < 0.05).

Exchangeable acidity was shown to be significantly higher in P + L fertilized soils and was significantly lower in control soil treatment. Control soils had the highest total cations while P + L had the lowest concentration of total cations (Table 1).

### Functional diversity of soil bacteria

Soil nutrient fertilization resulted in the variation in viability of the biogeochemical cycling bacteria. N cycling bacteria showed the highest colony-forming unit (CFU g^−1^) in control soils and lowest in P + L soils (Table 2). Species richness of N cycling bacteria was highest in + P soils while control soils showed decreased N cycling bacteria species richness (Table 2). N-fixing bacteria showed the highest CFU g^−1^ in + P soils while P + L soils had the lowest (Table 2). P-solubilizing bacteria showed the highest (CFU g^−1^) in control soils while P + L soils had the lowest (Table 2). P-solubilizing bacteria species richness was highest in + P soils, while P + L soils had the lowest. The increased CFU value indicates the viability of the bacteria for the respective biogeochemical cycles in these soils.

**Table 2:** The microbial community structural indices in soils supplemented with phosphorus and lime, soils rich in phosphorus and control soils collected at Ukulinga farm, KwaZulu-Natal.

Microbial-community structural indices	P + L	+ P	Control
**Nitrogen cycling bacteria**			
Richness	3	5	4
CFU/g	3.05 × 10^4^	2.98 × 10^6^	9.79 × 10^6^
Simpson diversity index (ƛ)	0.183	0.214	0.000
Shannon diversity index (H)	0.329	0.439	0.000
E_pielow_	0.695	0.517	1.000
R_margalef_	0.097	0.134	0.000
**Nitrogen fixing bacteria**			
Richness	2	3	1
CFU/g	3.33 × 10^6^	5.08 × 10^6^	3.57 × 10^6^
Simpson diversity index (ƛ)	0.461	0.399	0.544
Shannon diversity index (H)	0.654	0.589	0.883
E_pielow_	0.695	0.901	0.806
R_margalef_	0.067	0.065	0.133
**Phosphate-solubilizing bacteria**			
Richness	2	2	3
CFU/g	3.12 × 10^5^	6.98 × 10^6^	5.10 × 10^7^
Simpson diversity index (ƛ)	0.443	0.392	0.066
Shannon diversity index (H)	0.637	0.696	0.178
E_pielow_	0.631	0.401	0.299
R_margalef_	0.158	0.254	0.169

### Microbial species identity

The 16S ribosomal RNA gene partial sequence amplified from pure cultures showed that experimental soils had nitrogen cycling bacteria, nitrogen fixing bacteria and phosphate solubilizing bacteria. The identified N cycling bacteria included *Pseudomonas chlororaphis *subsp.* aurantiaca strain* PMR23O*, Pseudomonas spp.* BRJH1, *Pseudomonas monteilii strain* P36*, Pseudomonas fluorescens strain* B8*, Pandoraea oxalativorans strain* KSI 1495*, Pseudomonas koreensis strain* Y22*, Burkholderia contaminans strain J8A6SARS* and *Pseudomonas koreensis isolate* 2.SG.14 (Table 3). The identified N fixing bacteria included *Caulobacter rhizosphaerae strain* IMCC34905, *Sphingomonas* spp. N-9*, Pseudomonas putida isolate* P3_32A*, Pseudomonas koreensis isolate* 2.SG.14 and *Burkholderia contaminans strain* J8A6SARS (Table 4). The phosphate solubilizing bacteria included *Pseudomonas nitroreducens strain* HBP1, *Pseudomonas kribbensis strain* CHA-19, *Pseudomonas *sp.* Sampath* 10, *Pseudomonas stutzeri*, *Pseudomonas denitrificans* ATCC 13867, *Variovorax paradoxus strain* rif200835 and *Paenibacillus xylanilyticus strain* W4 (Table 5).

**Table 3:** The molecular identification of the isolated nitrogen cycling bacteria in soils supplemented with phosphorus and lime, soils rich in phosphorus and control soils collected at Ukulinga farm, KwaZulu-Natal.

Code	Strains	Accession no	Similarity (%)
A	*Pseudomonas chlororaphis* subsp. aurantiaca strain PMR23O	KY629627.1	98.92
B	*Pseudomonas* spp*.* BRJH1	KT888011.1	98.38
C	*Pseudomonas monteilii* strain P36	MW245839.1	95.68
D	*Pseudomonas fluorescens* strain B8	KF010368.1	98.62
E	*Pandoraea oxalativorans* strain KSI 1495	KC113145.1	98.84
F	*Pseudomonas koreensis* strain Y22	MN710458.1	98.48
G	*Burkholderia contaminans* strain J8A6SARS	MT409575.1	99.01
H	*Pseudomonas koreensis* isolate 2.SG.14	LR027418.1	98.20

**Table 4:** The molecular identification of the isolated nitrogen fixing bacteria in soils supplemented with phosphorus and lime, soils rich in phosphorus and control soils collected at Ukulinga farm, KwaZulu-Natal.

Code	Strains	Accession no	Similarity (%)
A	*Caulobacter rhizosphaerae* strain IMCC34905	MK 138628	97.38
B	*Sphingomonas spp.* N-9	LC 101917	97.82
C	*Pseudomonas putida *isolate P3_32A	LT 838135	98.48
H	*Pseudomonas koreensis* isolate 2.SG.14	LR 027418	98.20
J	*Burkholderia contaminans* strain J8A6SARS	MT 409575	99.01

**Table 5:** The molecular identification of the isolated phosphate solubilizing bacteria in soils supplemented with phosphorus and lime, soils rich in phosphorus and control soils collected at Ukulinga farm, KwaZulu-Natal.

Code	Strains	Accession no	Similarity (%)
A	*Pseudomonas nitroreducens strain* HBP1	CP 049140	98.51
B	*Pseudomonas kribbensis strain CHA-19*	MK 660005	90.10
C	*Pseudomonas *sp.* Sampath* 10	HM 749063	98.41
D	*Pseudomonas stutzeri*	FR 667889	99.65
E	*Pseudomonas denitrificans* ATCC 13,867	CP 004143	99.77
M	*Variovorax paradoxus* strain rif200835	FJ 527675	99.30
O	*Paenibacillus xylanilyticus* strain W4	CP 044310	85.41

**Figure 1:**
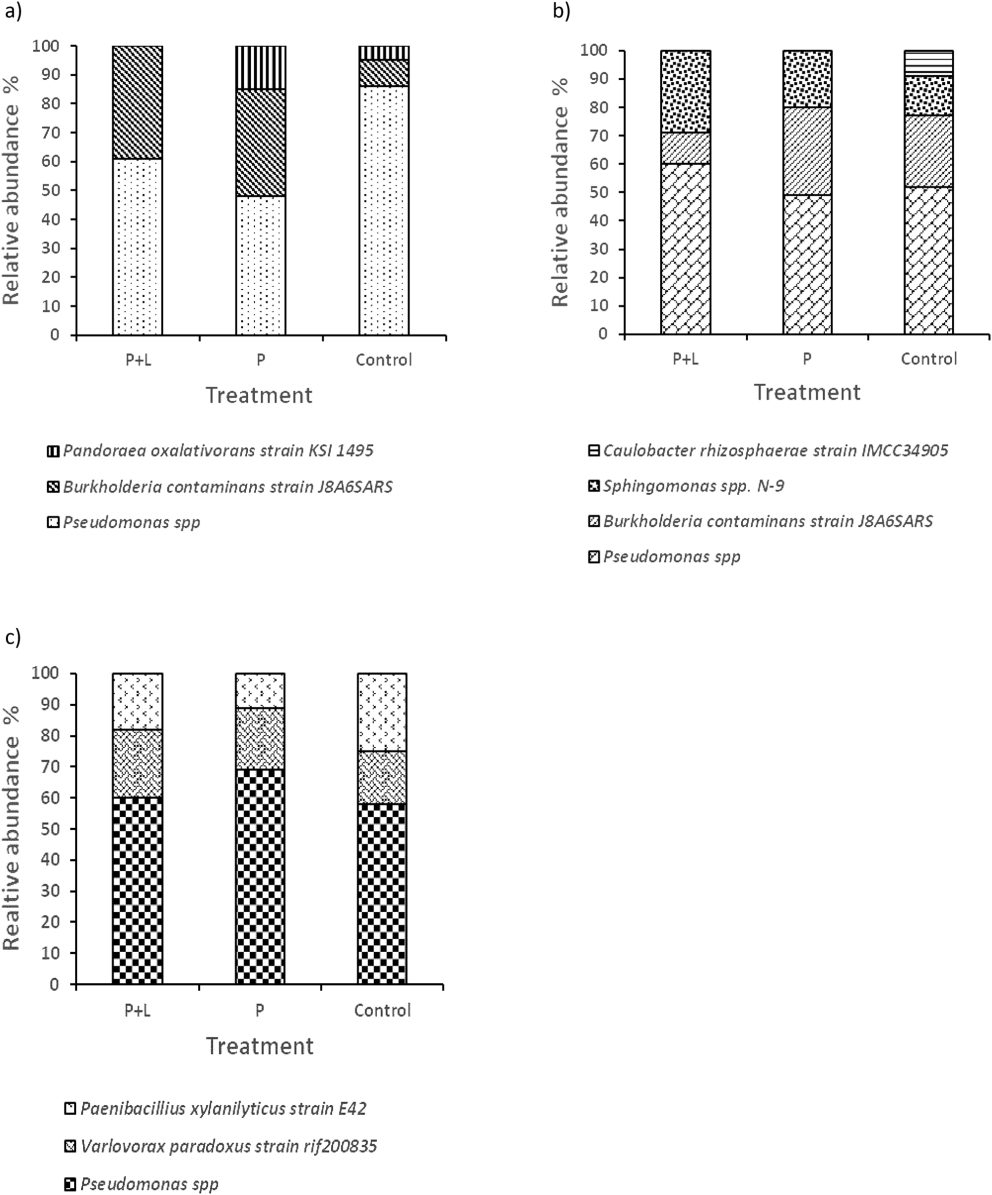
The relative abundance of the microbial communities (**a**) N cycling bacteria, (**b**) N fixing bacteria and (**c**) Phosphate solubilizing bacteria, soils supplemented with phosphorus and lime, soils rich in phosphorus and control soils collected at Ukulinga farm, KwaZulu-Natal.

*Pseudomonas* spp. had a high tolerance for nutrient variability and had an increased abundance in all the soil treatments (Fig. 1). Following *Pseudomonas* spp., *Burkholderia contaminans strain* and *Sphingomonas* spp. N-9 were able to tolerate more nutrient variability compared to other species (Fig. 1). *Caulobacter rhizosphaerae* was only unique in control soils.

### Extracellular enzyme activities

**Figure 2:**
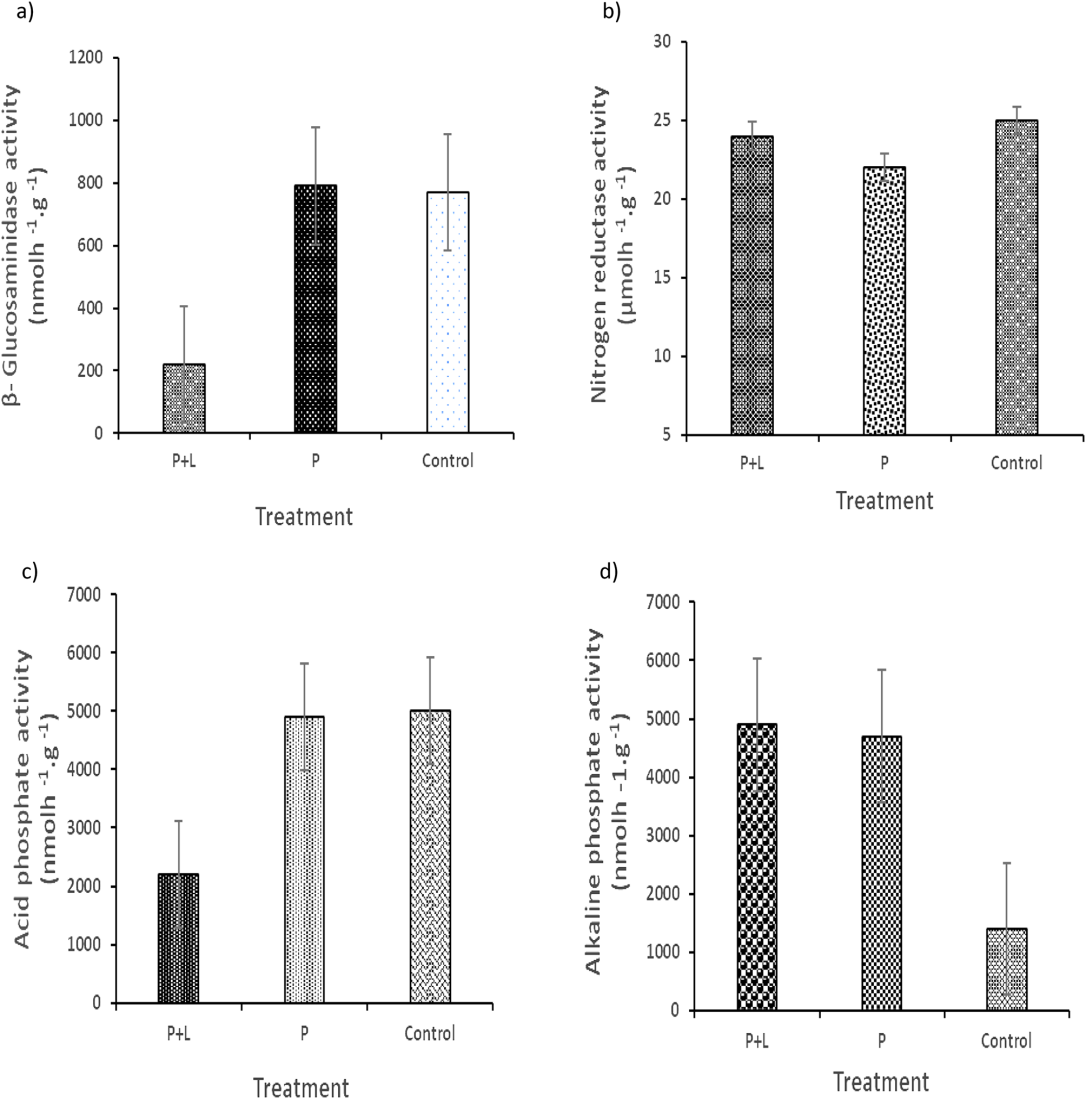
The effect of phosphate fertilization and liming on nitrogen and phosphate cycling enzymes; (**a**) β-Glucosaminidase activity, (**b**) N reductase activity, (**c**) Acid phosphate activity and (**d**) Alkaline phosphate activity.

β-glucosaminidase activity was significantly higher in + P and control soils and decreased activity in P + L soils (Fig. 2a), while N reductase activity was similar between the soil treatments (Fig. 2b). Acid phosphatase showed two-fold increased activity in control soils and + P soils compared to P + L soils (Fig. 2c). Alkaline phosphatase activity was three-fold higher in + P and P + L soils compared to the control soils (Fig. 2d).

### Correlation matrix graph

**Figure 3:**
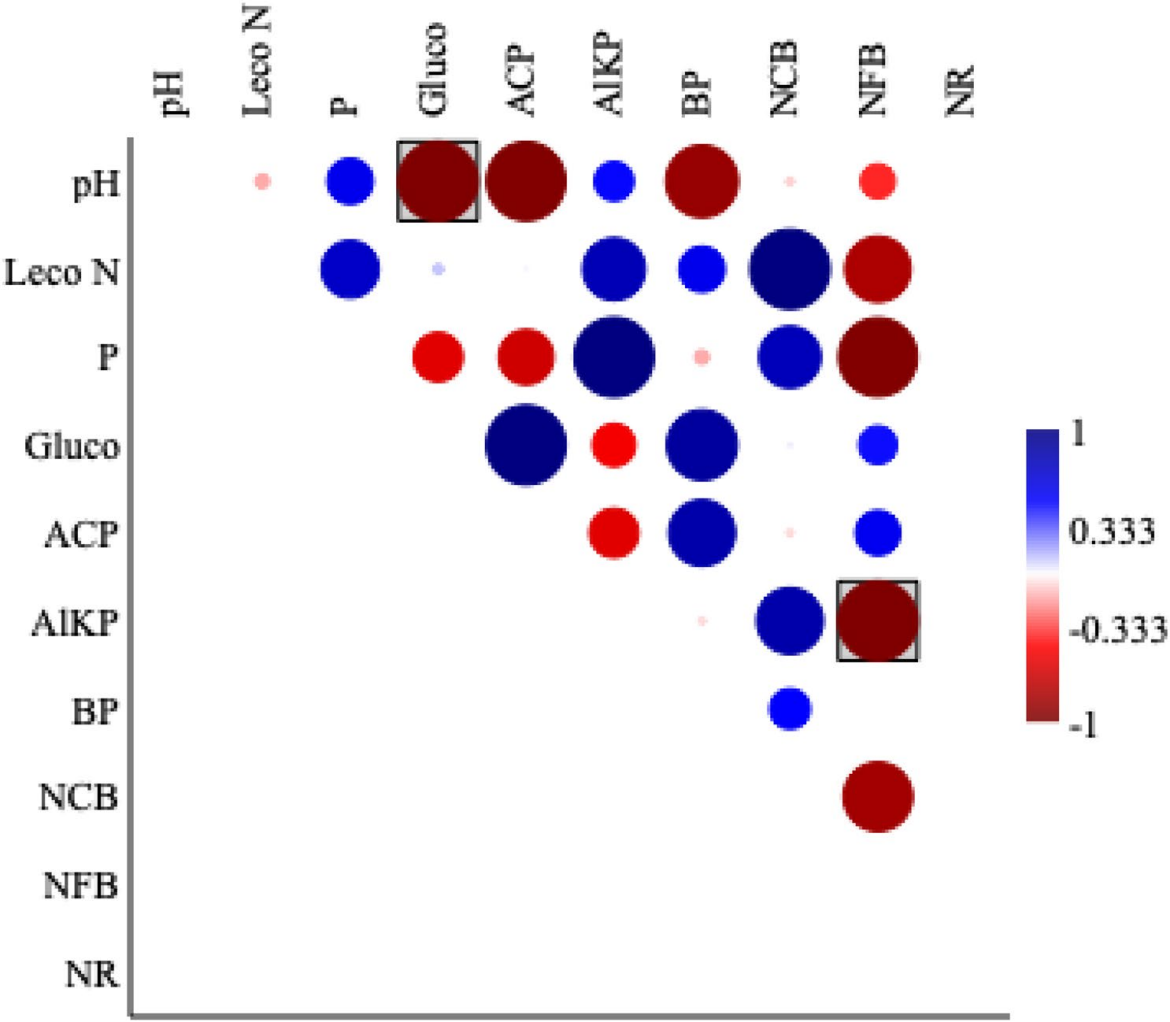
Linear correlations between pH, Inorganic N, inorganic P, N cycling enzymes, phosphate cycling enzymes and culturable bacteria communities. The red and blue circles represent negative and positive correlation respectively at (P < 0.05). The extent of correlation is indicated by pie fill area, i.e., larger to smaller pie fill area indicates high to low correlation. Key: β-gluco (β-glucosaminidase activity), NR (N reductase activity), ACP (Acid phosphate activity), ALKP (Alkaline phosphate activity), BP (Phosphate solubilizing bacteria), NCB (N cycling bacteria), NFB (N fixing bacteria).

Soil P concentrations and acid phosphatase activity showed a significantly negative correlation (P ≤ 0.05) (Fig. 3). While soil P concentrations and alkaline phosphatase activity had a significantly positive correlation (P ≥ 0.05). Acid phosphatase activity had a significantly negative correlation with alkaline phosphate activity. The soil P concentration showed a negative correlation with the N hydrolyzing enzyme (β-glucosaminidase). Acid phosphatase activity had a significant positive correlation (P ≥ 0.05) with β-glucosaminidase (Fig. 3).

## Discussion

Fertilizers are often used in enhancing soil fertility and plant productivity but could deeply modify soil properties and microbial communities over long term application^41^. Based on the soil geochemistry in our study, the high P, K, Ca, Mg, exchangeable acidity and total cations contents in P-fertilizer and liming soils (Table 1) in comparison with the control soil sample could be linked to alteration in the microbial communities as a result of long-term fertilization of the soil^42^. Likewise, the non-significant differences in organic carbon and nitrogen contents after prolonged fertilization are not an unusual occurrence in grassland ecosystems, owing to the fact that only organic fertilizer application does have an influence on increasing the pool of soil organic materials and nutrient availability^43^. Similarly, this observation was reported by Gautam^44^ where inorganic fertilizer could not play a vital role in the availability of labile fractions of C and N in the soil in comparison to where organic fertilizer (manure). Conversely, the slight increase in pH of P and liming-fertilized soil in comparison to other forms of fertilization could be attributed to the presence of CaCO_3_ in the lime (dolomitic), since dolomitic lime is known as a derivative from the deposits of calcium carbonate and magnesium carbonate with much higher levels of magnesium^45^. This observation is in agreement with the report of Xu^46^, during the validation of long-term fertilization impacts and liming on soil acidification modelling at Rothamsted experimental station in the United Kingdom, where liming was found to neutralized soil acidification. While the non-significant difference in the soil pH between the + P fertilized soil and the control is evidence of P is more strongly adsorbed by the soil with an implication of relatively less available for plant uptake^47^.

Several reports have documented that nutrient supplementation in soils causes a shift in the composition of plant communities with faster-growing plants that are good competitors for light being favoured under nutrient limiting conditions. While the information about the response of its accompanying belowground microbial communities such as general taxonomic and trait alterations, remains poorly understood, even though soil microbes represent a large fraction of the living biomass in grassland systems^48^. Thus, the presence of different microbial communities such as the culturable N-cycling, N-fixing and P solubilizing bacteria within various fertilization experimental soils suggest that long-term fertilization plays a major role in the regulation of microbial diversities, taxonomy and traits^43^. However, the high species richness of culturable bacteria (N-cycling and N-fixing bacteria) of + P fertilized soil in comparison to other fertilizations could be attributed to the utilization of P by the culture microbial communities for rapid growth with their metabolic activities since P is a major constituent during the production of ATP, RNA and DNA^49^. Interestingly, a slight reduction in species richness of + P + L fertilized soil in comparison with other fertilization agrees with the general hypothesis that soil microbial communities are sensitive to fertilization and it determines the specific functional microbial diversities^48^. Also, the slight reduction in species richness of the P-solubilizing bacteria could be linked to the presence of culturable bacteria belonging to the phyla Pseudomonadota and Bacillota respectively and capable of outcompeting other microbial communities in the soil ecosystem. For instance, the Pseudomonadaceae family from the Pseudomonadota are known as producers of a variety of secondary metabolites, many of which have activities such as antibiotics, enzymes, immunosuppressants, and plant growth regulators^50^. Members of the Paenibacillaceae family from the Bacillota are known to produce different classes of antibiotics, which can quickly and accurately kill various pathogens and some antibiotics that can enhance resistance to proteolytic enzymes^51^.

Microorganisms and extracellular enzymes in soils are important determinants of soil nutrition in ecosystems and agronomic practices^52^. Our study shows that enzyme activity changed with varying nutrient additions. The resource allocation model for extracellular enzyme activities in soil states that soil microorganisms produce enzymes for mineralization and cycling of insufficient soil nutrients^53^. In nutrient deficient soils, extracellular enzymes mineralize nutrients contributing to nutrient availability for plant uptake^54^. Phosphatase mineralize organic P compounds in the soil, as a result, phosphatase plays an important role in P cycling in P poor soils^55^. The enzymatic activities of acidic phosphatase and alkaline phosphatase are most active between pH values of 4 to 6 and 9 to 11, respectively^56^. The observed soil enzymes from our study shows that acid phosphatase enzymes increased activity in control soils. The increased activity of the acid phosphatase enzymes suggests that P in control soils may have been bound by cations and remained insoluble for plant uptake, requiring mineralization by acidic phosphatase. This was confirmed by the observed negative correlation between acid phosphatase enzyme activity and soil P concentration. In addition, control soils had increased activity of β-glucosaminidase enzymes, which also are involved in N cycling. β-glucosaminidase enzymes cycle N in soil by degrading proteins from organic matter, NH_3_ as well as amino acids^57^. As a result, their increased activity in control soils might have led to increased mineralization of N contributing to N cycling in the nutrient deficient grassland soils.

## Conclusion

The results from this study showed that long-term P fertilization and liming increased soil P concentration, soil pH, the presence of bacteria such as phosphate solubilizing-bacteria in KZN grassland soils. The presence of P-solubilizing bacteria, nutrient-solubilizing microbes and atmospheric N reducing bacteria plays a crucial role in nutrient cycling P available for plant uptake in this ecosystem. The abundance of the rhizobacteria (*Pseudomonadaceae*) suggest that they are the active producers of enzymes such as acid phosphatase and β-glucosaminidase during the nutrient cycling and mineralization process. Therefore, soil management practices that seek to address nutrient deficiency and soil acidity in nutrient stressed ecosystems may affect the functional role of soil microbes with their associated extracellular enzymes in these ecosystems.

## Data Availability

The datasets generated and/or analysed during the current study are available in ResearchGate under two different dataset files detailed below: [Soil microbes and associated extracellular enzymes largely impact nutrient bioavailability in acidic and nutrient poor grassland ecosystem soils molecular identification of the isolates, microbial diversity and enzyme activity data] repository, [https://www.researchgate.net/publication/361389402_Code_Strains] [https://www.researchgate.net/publication/361389722_microbial_diversity_and_enzyme_activity_data]”. Also, all raw data can be requested from the corresponding author, Dr Anathi Magadlela at anathimagadlela@icloud.com.
